# Lactate Metabolism and Immune Modulation in Breast Cancer: A Focused Review on Triple Negative Breast Tumors

**DOI:** 10.3389/fonc.2020.598626

**Published:** 2020-11-26

**Authors:** Adviti Naik, Julie Decock

**Affiliations:** Cancer Research Center, Qatar Biomedical Research Institute (QBRI), Hamad Bin Khalifa University (HBKU), Qatar Foundation (QF), Doha, Qatar

**Keywords:** triple negative breast cancer, lactate acidosis, immunotherapy, tumor metabolism, Warburg effect, metabolic reprogramming, anti-tumor immunity, immunosuppression

## Abstract

Triple negative breast cancer (TNBC) is an aggressive subtype of breast cancer associated with poor prognosis, early recurrence, and the lack of durable chemotherapy responses and specific targeted treatments. The recent FDA approval for immune checkpoint inhibition in combination with nab-paclitaxel for the treatment of metastatic TNBC created opportunity to advocate for immunotherapy in TNBC patients. However, improving the current low response rates is vital. Most cancers, including TNBC tumors, display metabolic plasticity and undergo reprogramming into highly glycolytic tumors through the Warburg effect. Consequently, accumulation of the metabolic byproduct lactate and extracellular acidification is often observed in several solid tumors, thereby exacerbating tumor cell proliferation, metastasis, and angiogenesis. In this review, we focus on the role of lactate acidosis in the microenvironment of glycolytic breast tumors as a major driver for immune evasion with a special emphasis on TNBCs. In particular, we will discuss the role of lactate regulators such as glucose transporters, lactate dehydrogenases, and lactate transporters in modulating immune functionality and checkpoint expression in numerous immune cell types. This review aims to spark discussion on interventions targeting lactate acidosis in combination with immunotherapy to provide an effective means of improving response to immune checkpoint inhibitors in TNBC, in addition to highlighting challenges that may arise from TNBC tumor heterogeneity.

## Introduction

Inter- and intra-tumor heterogeneity of breast tumors are a major causal factor for prognostic and drug response disparities. Among the breast cancer molecular subtypes, triple negative breast cancer (TNBC), accounting for 15–20% of all breast cancers, is defined by the absence of estrogen receptor (ER), progesterone receptor (PR), and human epidermal growth factor receptor-2 (Her-2) expression (1). TNBCs are particularly characterized by poor prognosis, early recurrence, and increased risk of metastasis, cumulatively accounting for 25% of all breast cancer-related deaths (2). In addition, the lack of hormone receptor expression renders TNBC tumors refractory to the targeted therapeutics currently being implemented for the treatment of hormone receptor positive breast cancer subtypes, essentially limiting treatment options to chemotherapy. Although TNBC tumors initially respond well to chemotherapy, they develop resistance and display early recurrence rates (3). In addition, the molecular heterogeneity within TNBCs has led to its classification into several intrinsic subtypes, further adding to the predicament of developing personalized approaches to treat TNBCs (4, 5).

Immunotherapy has revolutionized the treatment of several cancer types, particularly melanoma, lymphoma, renal cell cancer, and non-small cell lung cancer (6). This treatment modality involves activating the host immune system to recognize and eliminate tumor cells. Numerous types of cancer immunotherapy are being trialed and implemented, as reviewed in detail elsewhere (7). Immune checkpoint blockade (ICB) has progressed most prominently as an effective immunotherapy by targeting inhibitory T cell regulatory molecules such as programmed cell death-1 (PD-1), its ligands programmed cell death ligand 1/2 (PD-L1/L2), and cytotoxic T-lymphocyte associated antigen-4 (CTLA-4), thereby re-invigorating the anti-tumor immune response (8). In 2019, the US Food and Drug Administration (FDA) approved the use of Atezolizumab, a blocking antibody targeting PD-L1, in combination with nab-paclitaxel chemotherapy for first-line treatment of unresectable, PD-L1 positive, locally advanced, or metastatic TNBC. Although this is the only immunotherapy currently available to TNBCs, there are several clinical trials evaluating the efficacy of ICB in TNBCs as monotherapy or in combination with other treatment modalities (9).

Some key factors that influence the response to immunotherapy in solid tumors include the extent of tumor immune infiltration and the expression of immune checkpoint molecules. Within the breast cancer subtypes, TNBCs are considered to be the most immunogenic (10), in part due to higher levels of tumor-infiltrating lymphocytes (TILs), and higher tumor mutational burden and neoantigen load. Concordantly, TNBCs are enriched in the expression of immune checkpoint molecules, either on tumor cells or on infiltrating immune cells (11, 12). These properties provide rationale for the responsiveness of TNBCs to ICB compared to other breast cancer subtypes. Nonetheless, considering the heterogeneity of this subtype, only a small proportion of TNBCs indicate an immunomodulatory phenotype amenable to targeting with immunotherapy. Ali et al. reported that only 20% of TNBCs expressing core basal markers exhibit PD-L1 expression. Moreover, single-agent ICB response rates in unselected metastatic TNBC patient cohorts still remain low with limited durability (13).

Thus, improving the efficacy of immunotherapy in TNBCs requires a better understanding of factors that influence tumor immune infiltration and immune evasion. In this regard, tumor metabolism is known to play a critical role in shaping the tumor and immune microenvironment. Within the scope of this review, we will discuss the molecular factors driving the glycolytic nature of TNBCs, and explore their role in lactate-mediated modulation of the anti-tumor immune response. Finally, we will assess the clinical benefit of combining targeting of lactate metabolism with immune checkpoint blockade to improve the efficacy of immunotherapy in TNBCs.

## Metabolic Plasticity in TNBC

Under normal conditions, oxidative phosphorylation (OXPHOS) is the preferred mode of energy generation in somatic cells, including normal mammary epithelial cells. Particularly during lactation, glucose uptake is significantly increased in the mammary cells, the major proportion of which is metabolized to lactose in the Golgi apparatus (14). Under circumstances of oxygen deprivation, cells may switch from aerobic OXPHOS to glycolytic metabolism to reduce the generation of reactive oxygen species (ROS) and hence alleviate hypoxic stress (15). Likewise, rapidly dividing tumor cells rewire cellular metabolism to meet the high bioenergetic and anabolic demands of growing tumors in a nutrient-deprived microenvironment. This tumor characteristic or cancer hallmark is known as the ‘Warburg effect’, whereby tumors shift their metabolic preference from OXPHOS to aerobic glycolysis, even under oxygen-rich conditions (16). The shift to aerobic metabolism is thought to result from both intrinsic and extrinsic cues (17). Intrinsically, oncogenic mutations, aberrant expression of microRNAs and transcription factors, and cumulative mitochondrial defects in tumor cells instigate metabolic reprogramming (18–20). Extrinsic cues that promote metabolic reprogramming include reduced oxygen and nutrient availability, decreased extracellular pH, and microenvironment interactions with immune and stromal cells and the extracellular matrix (ECM). TNBC tumors often exhibit several of these features, rendering them more sensitive to metabolic reprogramming. TNBC cells show increased rates of glycolysis, as inferred from increased glucose uptake, overexpression of glycolytic enzymes, and increased oxygen consumption rate (OCR) and extracellular acidification rate (ECAR), in comparison to other breast cancer subtypes (21–23). Furthermore, TNBC cell lines display more glycolytic dependence compared to luminal breast cancer cell lines whereby treatment with the glycolysis inhibitor 2-deoxyglucose (2-DG) markedly reduced cell proliferation in TNBC cells (24). To gain insight into how the glycolytic nature of TNBCs may affect anti-tumor immunity and how this can be exploited for therapeutic purposes, it is important to identify the key molecules involved in the metabolic adaptation. In this context, we will explore any alterations in molecular determinants of glucose uptake, lactate to pyruvate interconversion, and lactate transport.

### Aberrant Expression of Glucose Transporters

The Warburg effect observed in tumors depends on the availability of glucose as a substrate. Glucose uptake into the cell is mediated by GLUT transporters, a family of transmembrane proteins, of which GLUT1 is the most widely expressed isoform in cancers, particularly in basal-like TNBC (25). In TNBC, GLUT1 overexpression correlates with higher histological tumor grade (26). Interestingly, silencing of GLUT1 in TNBC models reduces both cell proliferation and invasive potential, thus highlighting the role of GLUT1 and indirectly glucose scavenging in supporting the aggressive tumor behavior of TNBC (27). The expression of glucose transporters is regulated by c-Myc, a basic region helix–loop–helix leucine zipper (bHLHZip) transcription factor serving as a hub in regulating a broad range of cancer-related signaling pathways (28). Oncogenic mutations in c-Myc leading to overexpression are often observed in TNBC tumors whereby c-Myc functions antagonistically with MondoA, a nutrient-sensing transcription factor allowing cells, to adapt to changes in glycolytic flux (18, 29). Mechanistically, c-Myc upregulation in TNBCs directly suppresses the MondoA-dependent induction of thioredoxin-interacting protein (TXNIP), an inhibitor of glucose uptake and glycolysis, through competitive binding of the TXNIP promoter region (29). As TXNIP regulates the mRNA expression and protein stability of GLUT1, its suppression by c-Myc eventually results in enhanced glucose metabolism (30). In concordance, a Myc^high^/TXNIP^low^ signature correlates with poor clinical outcome in TNBC but not in non-TNBC subtypes (31). Moreover, this correlation was more prominent in the presence of p53 mutations which are frequently found in TNBC tumors, suggesting an indirect association between tumor mutation status and metabolism (19). Of interest, a familial genetics study reported a homozygous point mutation in the *TXNIP* gene that completely suppressed its expression, leading to lactate acidosis in the affected individuals (32). The presence of mutant TXNIP variants in breast cancer is yet unknown. Expression of GLUT1 can also be regulated through hypoxia response elements by hypoxia-inducible factor (HIF)-1a whose expression is correlated with *BRCA1* and basal phenotypes in breast cancer such as those observed in TNBC (33, 34). Another mechanism that supports GLUT1 stabilization, specifically in basal-like TNBC cells, involves the suppression of GLUT1 endocytosis and Akt-mediated degradation by the GTPase-activating protein USP6NL (35). Thus, TNBC tumors are intrinsically primed for enhanced glucose uptake to support their glycolytic phenotype. Although several long non-coding RNA, such as ANRIL and HOTAIR, have been shown to regulate GLUT expression in various tumor types, no reports are available yet for breast cancer (36).

### Upregulation of Lactate Dehydrogenases

Lactate dehydrogenases (LDHs) are key enzymes in glycolysis, regulating the interconversion of pyruvate to lactate. There are five L-lactate dehydrogenase isoforms that are composed of different combinations of LDH-M (M for muscle) and LDH-H (H for heart) subunits: LDH-1 (H4), LDH-2 (H3M1), LDH-3 (H2M2), LDH-4 (H1M3), and LDH-5 (M4) (37). The LDH-M and LDH-H subunits are encoded by the *LDHA* and *LDHB* genes and are alternatively denoted as LDHA and LDHB, hence, LDH-5 (M4) and LDH-1 (H4) are often referred to as LDHA and LDHB respectively. The LDH isoforms are associated with different tissue specificity with LDH-1/LDHB predominantly being expressed in the heart, LDH-5/LDHA in striated muscle, LDH-2 in the reticuloendothelial system, LDH-3 in the lungs, and LDH-4 in the kidneys. Additionally, there is a sixth isoform, LDHC or LDHX, that is composed of four LDHC subunits and is exclusively expressed in testis tissue (38). LDHA and LDHC preferentially catalyze pyruvate to L-lactate conversion, while LDHB has a higher affinity for lactate, thus collectively determining the rate of glycolysis.

In addition to their widespread expression in normal tissues, LDHA and LDHB are often overexpressed in tumor tissues, including TNBC. Furthermore, elevated circulating total LDH levels have been found to predict clinical outcome and treatment response to chemotherapy in advanced TNBC patients (39). LDHA expression is significantly upregulated in TNBC tumors compared to non-TNBC tumors and is associated with shorter overall- and disease-free survival (40). Increased tumoral and serum LDHA levels have also been correlated with brain metastasis and poor survival in patients with TNBC (41). In line with this finding, knocking down LDHA expression in the syngeneic 4TI TNBC mouse model decreased tumor-derived lactate levels, tumor growth rate and metastases (42). LDHB is also upregulated in TNBC (24) and PAM50 basal-like subtypes (43). The function of LDHB in breast cancer or more specifically TNBC remains ambiguous. The role of LDHB in promoting lysosomal acidification required for autophagy-associated vesicle maturation and protease activation has been reported as a mechanism by which LDHB can promote tumor cell proliferation and survival in some cancer types (44). High LDHB expression in basal-like breast cancer has been associated with better pathological complete response rates to neoadjuvant chemotherapy (43). LDHB has been reported to complement the role of LDHA in colon adenocarcinoma and melanoma models with metabolic pressure (45). More specifically, knockout of both LDHA and LDHB was required to suppress glycolysis under hypoxic conditions and hence, curb tumor growth, but under normoxic conditions the tumor cell metabolism shifted to OXPHOS as an energy source. Although the substrate preference of LDHA and LDHB differs, these observations indicate that substrate affinity and the extent of metabolic adaptation in tumors may vary depending on both tumor-specific intrinsic and extrinsic cues. The LDHC isoenzyme is an immunogenic germline-specific antigen that is re-expressed in a wide variety of cancer types (46, 47). Particularly, high levels of circulating LDHC in serum and tumor-derived exosomes are negatively correlated with breast cancer prognosis (48). Expression of LDHC has been reported to play a role in propagating TNBC tumor cell invasion and migration (49). To date, LDHC has been implicated in glycolysis and energy metabolism of sperm only (50).

From the current literature, LDHA appears to be a key enzyme in TNBC-associated lactate acidosis. Studies in different cancer types have reported that LDHA overexpression stems from mechanisms involving transcriptional, post-transcriptional, and post-translational regulation (37). For instance, HIF-1a, c-Myc and the forkhead box M1 (FOXM1) transcription factor have been shown to bind to the LDHA promoter region to regulate its transcription (51). However, it remains to be understood if these regulatory mechanisms are ubiquitous across different cancers or alternative modes of regulation exist in TNBC. Moreover, the metabolic role of LDHB and LDHC in TNBC require thorough investigation.

### Dysregulation of Lactate Transport and Metabolic Symbiosis

The concentration of lactate in solid tumors has been reported to be chronically high (up to 50 mM) in comparison to physiological levels in the blood (up to 2 mM) (15). Quantification of lactate concentration in freshly excised tumors from a small cohort of 30 breast cancer patients using double quantum filtered magnetic resonance spectroscopy indicated that a higher tumor grade was associated with increased lactate concentration (52). A trend of increased mean lactate concentration (8.4 mM) was also reported in a small group of six TNBC tumors compared to nine non-TNBC tumors (7.2 mM) in the same study, however this observation needs confirmation in a larger population. Lactate was initially thought to be a mere waste metabolite from aerobic glycolysis. However, we now know that lactate has an active tumorigenic role as a biosynthetic precursor, signaling molecule and regulator of extracellular acidosis, and has therefore been referred to as an “oncometabolite” (53). Considering the excessive rate of glycolysis in tumors, the intracellular concentration of lactate can accumulate rapidly, serving as a rate-limiting step within the glycolysis pathway and impairing enzymatic function and cell proliferation. To avoid excessive pools of intracellular lactate, lactate is transported across the plasma membrane by the monocarboxylate family of transporters (MCTs) that are encoded by solute carrier 16 (*SLC16*) genes. Among these transporters, MCT1 (SLC16A1) and MCT4 (SLC16A3) have been extensively characterized in multiple tumors (54). MCTs are passive symporters transporting lactate anions in conjunction with protons, implying their function in equilibrating the lactate concentration and pH gradient across the intra- and extra-cellular compartments. Generally, MCT1 is involved in lactate import or export depending on the cell type and context while MCT4 primarily functions in lactate efflux from glycolytic cells into the microenvironment.

**Figure 1:**
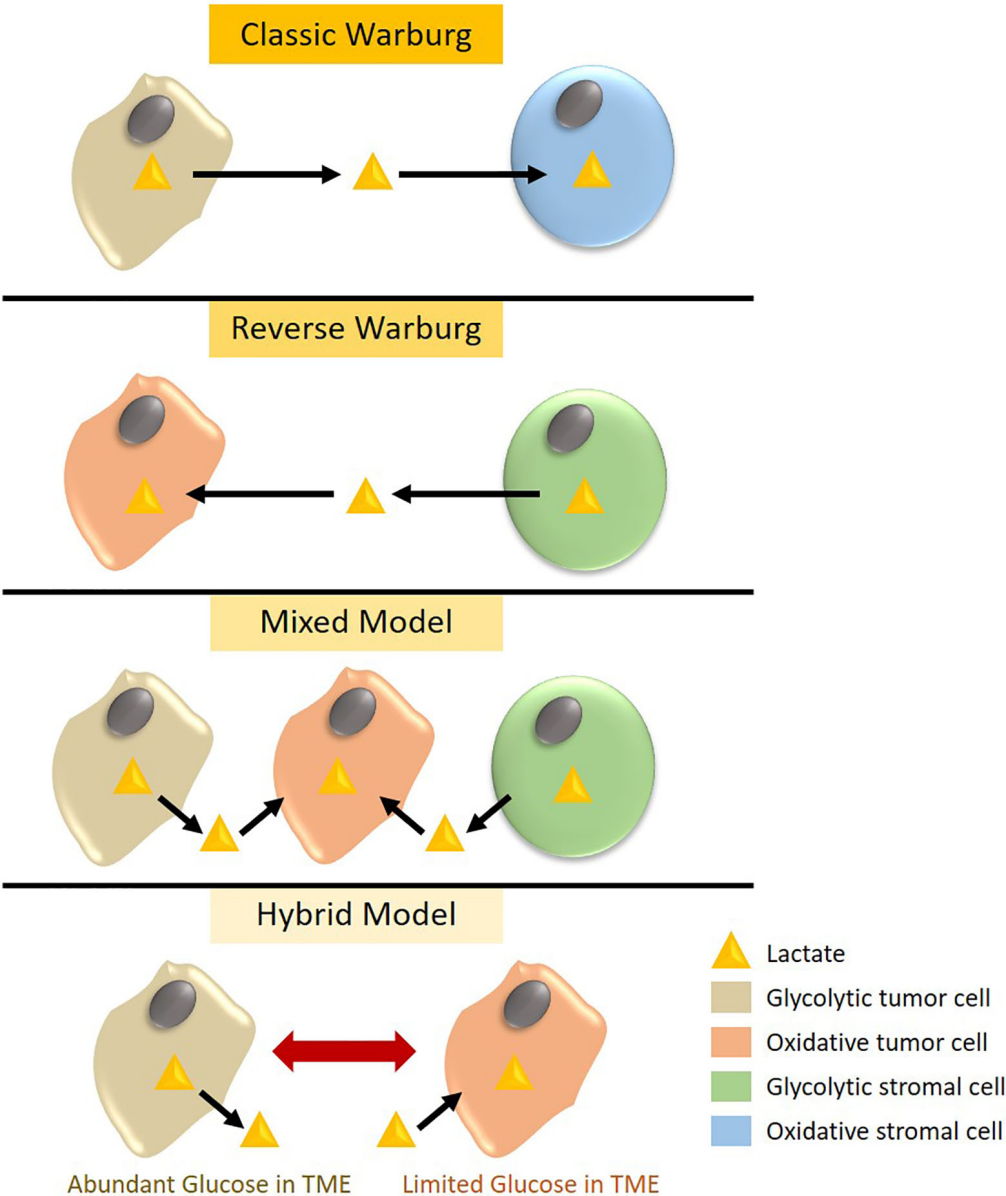
Metabolic phenotypes observed in triple negative breast cancer (TNBC). According to the classic Warburg theory, glycolytic TNBC cells expressing high levels of the lactate transporter MCT4 export lactate, which is taken up by MCT1-expressing stromal cells to generate energy through oxidative phosphorylation (OXPHOS). Alternatively, MCT4 expressing glycolytic stromal cells can export lactate that is used by oxidative tumor cells in a phenomenon called the reverse Warburg effect. The mixed model represents metabolic symbiosis in heterogeneous tumors whereby glycolytic tumor and stromal cells generate lactate to feed oxidative tumor cells. Lastly, the hybrid model depicts metabolic plasticity in TNBC tumor cells that can switch between a glycolytic and oxidative phenotype based on extrinsic cues and glucose availability in the tumor microenvironment (TME).

According to the Warburg effect, tumor cells undergo a metabolic switch to aerobic glycolysis whereby glycolytic tumor cells expressing MCT4 export lactate and oxidative tumor cells or stromal cells with high MCT1 expression import lactate to use as an energy source through OXPHOS. In contrast, the reverse Warburg effect offers a state of metabolic symbiosis with reciprocal interactions between tumor and stromal cells, whereby glycolytic stromal cells provide lactate as a fuel to oxidative tumor cells (55). The existence of the reverse Warburg effect in TNBC tumors is under debate with some studies advocating for the traditional Warburg effect or a mixed model while others provide experimental evidence for the presence of the reverse Warburg phenotype (**Figure 1**). In support of the former, an immunohistological study by Choi et al. classified 740 breast cancer cases into different metabolic subgroups based on the expression of metabolic markers such as GLUT1 and MCT4 (56). Tumors were either considered to be of the Warburg type (glycolytic tumor cells and non-glycolytic stromal cells), reverse Warburg type (non-glycolytic tumor cells and glycolytic stromal cells), mixed type (glycolytic tumor cells and stromal cells), or null type (non-glycolytic tumor cells and stromal cells). Based on this classification, the majority of TNBC tumors displayed a Warburg or mixed metabolic phenotype, both characterized by high MCT4 expression, while luminal-type breast tumors mainly belong to the reverse Warburg or null metabolic phenotype, consistent with their metabolically inactive and less aggressive clinical presentation. In accordance, MCT4 expression strongly correlates with worse survival in TNBC as compared to luminal-type breast cancer (23, 56). TNBC tissue microarrays indicated that basal-like TNBC tumors in particular expressed glycolysis markers such as GLUT1 and MCT4, whereas non-basal-like TNBCs were represented by a glutaminolysis or mitochondrial metabolism phenotype (57). Furthermore, MCT4 ablation in the TNBC cell line MDA-MB-468 reduced cell viability and lactate secretion, enhanced OXPHOS, sensitized cells to mitochondrial respiration inhibitors, and impeded orthotopic tumor growth (58).

In support of the reverse Warburg phenotype, Witkiewicz et al. identified that MCT4 expression in stromal cells, but not tumor cells, was associated with poor survival in TNBC (59). In addition, loss of stromal caveolin-1, an indicator of hypoxia, has been associated with selective MCT4 stromal and MCT1 tumor expression and poor clinical outcome in TNBC (60). Combining positive stromal MCT4 with negative stromal Caveolin-1 expression improved stratification of TNBC cases with a high risk of recurrence and metastasis. Moreover, MCT1 expression in tumor cells showed a strong positive correlation with LDHB expression in TNBC tumors, corroborating the presence of the reverse Warburg effect (24). More specifically, basal-like TNBC tumors demonstrate increased MCT1 expression that is associated with a high proliferative index and histological grade (61). Of note, silencing of MCT1 in basal-like TNBC models disrupted lactate export and tumor growth *in vivo* (62), suggesting that MCT1 can adapt for bidirectional lactate transport in tumors.

In addition to the classical Warburg, reverse Warburg and mixed metabolic phenotype models, few studies have suggested the existence of a hybrid metabolic state in TNBC tumors and metastatic lesions (**Figure 1**) whereby tumor cells exhibit both high glycolytic and OXPHOS activity, allowing these tumors to switch between metabolic phenotypes for their bioenergetic demands in response to microenvironmental cues (63, 64). Targeting both glycolysis and OXPHOS in metastatic TNBC cells was required to eliminate this metabolic plasticity and hence, reduce their proliferation and survival.

Mechanistically, elevated MCT1 in TNBCs has been attributed to low levels of its regulatory miRNA miR-342-3p (65). In addition, the stability and localization of MCT1 and MCT4 are regulated by the chaperone glycoprotein CD147 that is upregulated in TNBCs compared to other breast cancer subtypes. CD147 expression is directly correlated with high tumor grade, basal markers, shorter progression-free and overall survival, and poor response to chemotherapy in TNBC (66, 67). Furthermore, the lactate sensing G-protein-coupled receptor 81 (GPR81), also known as hydrocarboxylic acid receptor 1 (HCAR1) has been implicated in an autocrine feedback loop that regulates MCT1 and/or MCT4 expression and their chaperone CD147 (68–70). GPR81 is highly expressed in many tumor types including breast cancer, in particular hormone receptor positive breast cancers where it is associated with improved overall survival and lower risk of distant metastasis (69, 71, 72). Silencing of GPR81 in hormone receptor positive breast tumor cells reduced the expression of specifically MCT1 but not of MCT2 or MCT4, resulting in decreased lactate uptake, extracellular acidification, and inhibition of tumor cell proliferation and survival (69). Hence, GPR81 may support the OXPHOS phenotype in these breast tumors by sensing and regulating influx of extracellular lactate. However, the role of GPR81 in TNBC-associated lactate signaling has not yet been reported and mandates future investigation. It is plausible that the high levels of lactate in the TNBC micromilieu constitutively activate GPR81, resulting in a negative feedback loop yielding reduced levels of GPR81 in glycolytic TNBC tumors. Alternatively, additional previously unidentified lactate-sensing GPCRs may play a role in TNBCs. Expression of GPR81 in tumor cells can be regulated through an autocrine feedback loop of lactate by the induction of Signal transducer and activator of transcription 3 (STAT3) that directly binds to the GPR81 promoter to induce its expression (73). Interestingly, lactate-induced expression of GPR81 has been shown to trigger the tumor expression of the immune checkpoint ligand PD-L1, indicating an additional dimension of lactate-mediated immune dysregulation in the tumor milieu to dampen anti-tumor immunity (74), as will be discussed in the following section.

## Lactate-Rich Environment Mediates Immunosuppression

Normal mammary gland architecture comprises of diverse cell types, including immune cells, which are essential at various stages of mammary organogenesis (75, 76). During malignant transformation, the mammary gland undergoes considerable reorganization of the tissue architecture as well as changes in cellular composition and cellular properties (77). Likewise, the tumor microenvironment of breast tumors is comprised of numerous cell types, including tumor cells, cancer-associated fibroblasts, various cell types forming vascular networks and immune cells. The composition and functionality of this complex landscape is ultimately shaped by a network of interacting extracellular cues such as lactic acid, subsequently influencing the anti-tumor immune response (**Table 1**) (93). Here, we will specifically discuss lactate-mediated changes in anti-tumor immunity in TNBC, focusing on pro-inflammatory immune cell subsets such as T lymphocytes, natural killer cells, dendritic cells, as well as immune suppressive myeloid-derived suppressor cells, T regulatory cells, and tumor-associated macrophages.

**Table 1:** Impact of lactate acidosis on immune cells in the tumor microenvironment.

Immune cell	Effect of lactate acidosis	References
**T lymphocytes**	- Diminished lactate export - Decreased glycolysis, proliferation, and cytotoxicity - Inhibited expression of IFN-γ and IL-2 cytokines - Enhanced mitochondrial dysfunction and ROS production - Increased apoptosis - Polarization to iTregs	(78–80)
**NK cells**	- Decreased tumor infiltration, proliferation, and cytotoxicity - Inhibited expression of activation receptors NKG2D and NKp46 - Dampened expression of IFN-γ, perforin, and granzyme - Enhanced mitochondrial dysfunction and ROS production - Impaired proliferation and differentiation of NKT cells	(81–83)
**DCs**	- Lactate sensed by GPR81 and imported by MCTs - Decreased glycolysis - Hindered maturation, activation, and antigen presentation - Impaired priming of T cells - Inhibited expression of IFN-α, IL-6, and IL-12 cytokines - Upregulated expression of IL-10 - Increased production of kynurenine that induces Tregs	(72, 84, 85)
**MDSCs**	- Increased proliferation and immunosuppressive activity - Induced development by tumor-derived G-CSF and GM-CSF	(81, 86)
**Tregs**	- Metabolic adaptation to suppress glycolysis and increase OXPHOS - Increased survival and proliferation	(87, 88)
**Monocytes**	- Diminished lactate export - Decreased glycolysis - Inhibited expression of IFN-γ and TNF-α cytokines - Upregulated expression of IL-17 and IL-23 cytokines	(89, 90)
**TAMs**	- Lactate sensed by GPR132 and imported by MCTs - Polarization from M1 to anti-inflammatory/pro-tumorigenic M2 - Increased OXPHOS - Upregulated expression of pro-tumorigenic ARG1, VEGF, and CCL5 - Enhanced secretion of immunosuppressive cytokines that subdue TIL cytotoxicity and promote Treg induction	(91, 92)

ARG1, arginase 1; CCL5, CC chemokine ligand 5; DCs, dendritic cells; G-CSF, granulocyte colony-stimulating factor; GM-CSF, granulocyte macrophage colony-stimulating factor; GPR, G-protein receptor; IFN-γ, interferon gamma; IL, interleukin; MCT, monocarboxylate transporters; MDSC, myeloid-derived suppressor cells; NK, natural killer cells; NKG2D, natural killer group 2 member D; NKT, natural killer T cells; OXPHOS, oxidative phosphorylation; ROS, reactive oxygen species; TAMs, tumor-associated macrophages; TIL, tumor infiltrating lymphocyte; TNF- α, tumor necrosis factor alpha; Treg, T regulatory cell; VEGF, vascular endothelial growth factor.

### T Lymphocytes

The number of tumor infiltrating lymphocytes (TILs) has consistently been identified as a prognostic and predictive biomarker in early stage TNBC (94). However, as tumors progressively grow larger, metabolic competition ensues and impairs the activity of various immune cell subpopulations (95). Cytotoxic CD8+ lymphocytes (CTLs) profoundly rely on glycolysis for proliferation and activation of their effector function (96). Thus, high rates of glycolysis in TNBCs offer a competitive advantage for tumor cells by restricting cytotoxic T cell metabolism and functionality. In addition, there is a feedforward mechanism whereby the lactate-rich environment of glycolytic tumors interferes with lactate export in cytotoxic T cells, which depends on an active lactate gradient, therefore resulting in increased intracellular lactate levels that inhibit metabolism, proliferation, and production of interferon (IFN)-γ (78). In concordance, Lim et al. observed that epidermal growth factor receptor (EGFR) signaling in TNBC cells and murine models promoted aerobic glycolysis and lactate efflux, subsequently dampening the activation of CTLs and the production of IFN-γ and interleukin (IL)-2 (79). Similar observations have also been reported for highly glycolytic melanomas, wherein LDHA^high^ tumors dampen IFN-γ-producing CD8+ T cells due to lactate acidosis (80). Conversely, reducing LDHA-mediated lactic acid production has been found to enhance T cell-mediated tumor killing, improve IFN-γ-producing T cell infiltration, and reduce melanoma tumor size (97–99). Furthermore, tumor-derived lactate enhances mitochondrial dysfunction and excess ROS production in naïve T cells, leading to apoptosis, by a mechanism involving the inhibition of focal adhesion kinase (FAK) family-interacting protein of 200 kDa (FIP200), a suppressor of the pro-apoptotic Bcl-2 family of proteins (100).

Molecular mechanisms driving this phenomenon involve the ability of lactic acid to inhibit IFN-γ transcription by preventing the upregulation of nuclear factor of activated T cells (NFAT), which is required for T cell and natural killer (NK) cell activation (80). Additionally, suppressed IFN-γ production has been linked to diminished mitogen-activated protein kinase (MAPK)/p38 and c-Jun N-terminal kinase (JNK) activity stemming from impaired T-cell receptor (TCR) activation under conditions of lactate acidosis in tumors (97). Finally, lactate acidosis, resulting from increased release of protons during lactate transport, has been shown to directly affect CTL cytolytic activity, cytokine secretion, and TCR activation, by lowering the pH in the tumoral niche (101).

### Natural Killer Cells

NK cells are innate effector lymphoid cells with anti-tumor cytolytic activity that is orchestrated by the secretion of pro-inflammatory cytokines and cytotoxic granules. In TNBC, NK cell infiltration has been associated with improved survival (102, 103). The inhibitory effect of lactate on NK cell cytotoxic activity has been reported for numerous cancers and involves downregulation of the expression of IFN-γ, perforin, granzyme, and the activating receptor NKp46 (81, 104). In line with this observation, glycolytic melanomas with high LDHA expression and lactate secretion show reduced NK cell activity and infiltration (80). In breast cancer specifically, tumor-infiltrating NK cells display decreased expression of the NKG2D activating receptor as compared to their counterparts in normal tissue (82). Inhibition of the lactate transporter MCT1 in the syngeneic 4T1 TNBC mouse model reduced lactate efflux and tumor growth, accompanied by an increased frequency of NKG2D/perforin/CD107a-expressing NK cells with improved cytotoxicity. Lactate-rich colorectal cancer liver metastasis exhibits a scarcity of NK cells with mitochondrial dysfunction and excessive ROS production leading to apoptosis, which could be recapitulated by treating healthy liver resident NK cells with lactic acid *in vitro* (105).

Invariant NKT cells, with properties of both NK and T cells, can also elicit an anti-tumor immune response by rapidly producing pro-inflammatory and immunomodulatory cytokines and cytotoxic perforin/granzyme B granules. Activation of NKT cells entails glucose uptake *via* the GLUT1 transporter and a glycolytic switch in metabolism, which is dependent on mTOR complex (mTORC) signaling (106). Exposure to high lactate levels inhibits NKT survival and proliferation. Mechanistically, acidosis induced by tumor-derived lactic acid inhibits the mTOR pathway and nuclear translocation of promyelocytic leukemia zinc-finger (PLZF), a regulator of NKT expansion and functional differentiation, resulting in impaired production of IFN-γ and IL-4 (83). The role, functional status, and prognostic value of NKT cells in TNBC remain to be investigated.

### Dendritic Cells

Dendritic cells (DCs) are a specialized class of antigen presenting cells involved in antigen processing and cross-presentation to CD8+ T cells. DC-mediated tumor rejection has been attributed to their ability to sense tumor-derived nucleic acids and activation of the type-I IFN system. Similar to CTLs, DCs rely on a metabolic switch from OXPHOS to glycolysis for activation, thus potentially ensuing metabolic competition within the tumor microenvironment (107). Lactic acid was shown to impair DC maturation, activation, cross-presentation, type-I IFN response, and antigen degradation (84, 108). In a syngeneic 4T1 TNBC mouse model, MCT-mediated lactate uptake by plasmacytoid DCs (pDCs), natural type I interferon–producing cells with antigen-presenting potential, inhibited their glycolysis capacity and thus IFN-α production while inducing the production of tryptophan-derived kynurenine and subsequent proliferation of T regulatory cells (Tregs) (85). In addition, GPR81 expressed on pDCs senses extracellular lactate and mobilizes intracellular calcium, which further has an inhibitory effect on DC activation and IFN-α expression. Lactate-dependent acidosis also inhibits DC differentiation through the induction of IL-10 production with concomitant loss of IL-12 (109). Similarly, lactate-mediated activation of GPR81 in DCs was found to abrogate antigen presentation, secretion of pro-inflammatory cytokines IL-6 and IL-12 and T cell function, and was associated with increased tumor growth in murine breast cancer models (72). In line with these findings, one study reported a high frequency of tumor-derived DCs with suppressed IFN-α production in aggressive, highly proliferative TNBC tumors, enabling the sustenance and expansion of Tregs and priming of anti-inflammatory IL-10-secreting CD4+ T cells (110).

Thus, the lactate-induced tolerogenic phenotype of tumor-infiltrating DCs indirectly impacts the priming of T lymphocytes and promotes an immunosuppressive cytokine profile and Treg expansion, collectively reinforcing tumor immune escape.

### Myeloid-Derived Suppressor Cells

Myeloid-derived suppressor cells (MDSC) are immunosuppressive immune cells that restrict T cell function, proliferation, and TCR signaling, and promote differentiation of Tregs (111). In TNBC tumors, glycolytic gene expression profiles (including *LDHA*) correlate with MDSC gene signatures and both associate with reduced survival (86). Increased glycolysis and hence lactate production was found to induce MDSC development and immunosuppression in murine TNBC models through the activation of the LDHA/AMP-activated protein kinase (AMPK)- Unc-51 Like Autophagy Activating Kinase 1 (ULK1)/autophagy axis, thereby promoting the expression of granulocyte colony-stimulating factor (G-CSF) and granulocyte macrophage colony-stimulating factor (GM-CSF) (86). Conversely, glycolytic restriction enhanced T cell immunity, reduced tumor growth and metastasis, and prolonged survival in the TNBC murine model (86). Depletion of LDHA to lower lactate production also decreased the frequency and immunosuppressive activity of MDSCs in a highly glycolytic murine pancreatic tumor model (81). This effect was directly attributed to lactate-mediated induction of MDSC proliferation, as observed by *in vitro* experiments supplementing lactate to human peripheral blood mononuclear cell co-cultures. Interestingly, as MDSCs rely on glycolysis for proliferation and their immunosuppressive activity by evading ROS-mediated apoptosis and enhancing mTOR pathway activation (112, 113), it remains to be understood how MDSCs thrive with metabolic competition in glycolytic tumor environments.

### T Regulatory Cells

Immunosuppressive Tregs undergo metabolic adaptation in low-glucose, lactate-rich tumor microenvironments. Specifically, an upregulation of the Treg-specific transcription factor forkhead box P3 (FOXP3) mediates induction of OXPHOS, alongside suppression of c-Myc expression and glycolysis (87). This metabolic reprogramming in Tregs, which are particularly enriched in TNBC tumors (114), makes them less dependent on glycolysis and enables the cells to efficiently turnover lactate into pyruvate. In addition, an increased nicotinamide adenine dinucleotide (NAD):NADH ratio in Tregs compensates for the lack of glycolytic activity and hence, renders them resistant to the inhibitory anti-glycolytic effects of lactate observed in T cells, and can polarize conventional T cells into induced Tregs (iTregs) that thrive on the metabolic symbiosis with glycolytic tumor cells (88).

### Tumor-Associated Macrophages

Tumor-associated macrophages (TAMs) are abundant in tumors, wherein extracellular stimuli guide their polarization between the pro-inflammatory “M1” subtype and anti-inflammatory “M2” subtype (115). In comparison to the glycolytic metabolism in M1 macrophages, M2 TAMs rely on OXPHOS to meet their bioenergetic demands—a trait that may additionally support the metabolic symbiosis between highly glycolytic TNBC tumor cells and M2 TAMs (116). Indeed, several studies have reported that M2 TAMs in TNBC tumor stroma positively associate with higher grade, larger tumor size, and poor survival whereas an inverse correlation has been observed in luminal A breast tumors that primarily depend on OXPHOS (117, 118). Moreover, co-culturing monocytes (precursors of macrophages and DCs) with the TNBC cell line MDA-MB-231 induced an M2 macrophage phenotype (119). Taken together, it can be envisaged that the lactate-rich landscape in TNBC tumors drives re-education of TAMs to an M2 phenotype. Indeed, tumor-derived lactate can induce TAM polarization to the M2 immunosuppressive phenotype by binding to the lactate-sensitive receptor GPR132 (91, 120). In turn, the M2 TAMs promote breast tumor cell migration and invasion *in vitro* and metastasis *in vivo*, thus supporting a positive feedback loop between tumor cells and pro-tumorigenic M2 TAMs. Concordantly, high GPR132 expression in breast cancer tumors correlated with the expression of M2 macrophage markers and low metastasis-free and relapse-free survival. Moreover, abrogating the lactate/GPR132 axis impedes M2 polarization and breast cancer metastasis in mice. In addition to lactate sensing by GPR132, lactate uptake by MCTs in TAMs also mediates M2 polarization (91). Lactate-induced TAM polarization and its pro-tumorigenic effects in breast cancer has been attributed to TAM-specific extracellular signal-regulated kinase (ERK)/STAT3 activation, stimulated expression of vascular endothelial growth factor (VEGF) and arginase-1 (ARG1), and stabilization of HIF-1a (91, 121, 122). Another mode of lactate-associated paracrine signaling between TAMs and breast tumor cells was reported by Lin et al., who showed that tumor cell-derived lactate induced the Notch pathway in TAMs to generate CC chemokine ligand 5 (CCL5) which then binds to its receptor CCR5 on breast tumor cells to promote aerobic glycolysis, migration, and epithelial-to-mesenchymal transition (EMT) (123). Besides driving tumorigenesis, M2 macrophages also secrete immunosuppressive cytokines that subdue the cytotoxicity of TILs and promote the differentiation of Tregs (92).

Furthermore, elevated levels of extracellular lactate prevent the expulsion of lactate generated in macrophage precursor monocytes, prompting a negative feedback mechanism for glycolysis and tumor necrosis factor (TNF) release (89). In toll-like receptor (TLR)-activated monocytes, lactic acid was also observed to induce the IL-23/IL-17 pathway, thus polarizing the immune response towards a pro-tumorigenic Th17 profile while suppressing the anti-tumor Th1 response (90, 124). Consistent with this observation, Th17 cytokines are upregulated in TNBCs compared to other breast cancer subtypes, especially in “immune-cold” tumors that are devoid of TILs (125). Thus, lactate imposes adverse effects on not only macrophage function and polarization, but also on its precursor monocytes.

## Targeting Lactate-Mediated Immune Evasion in TNBC: Potential Strategies and Challenges

**Figure 2:**
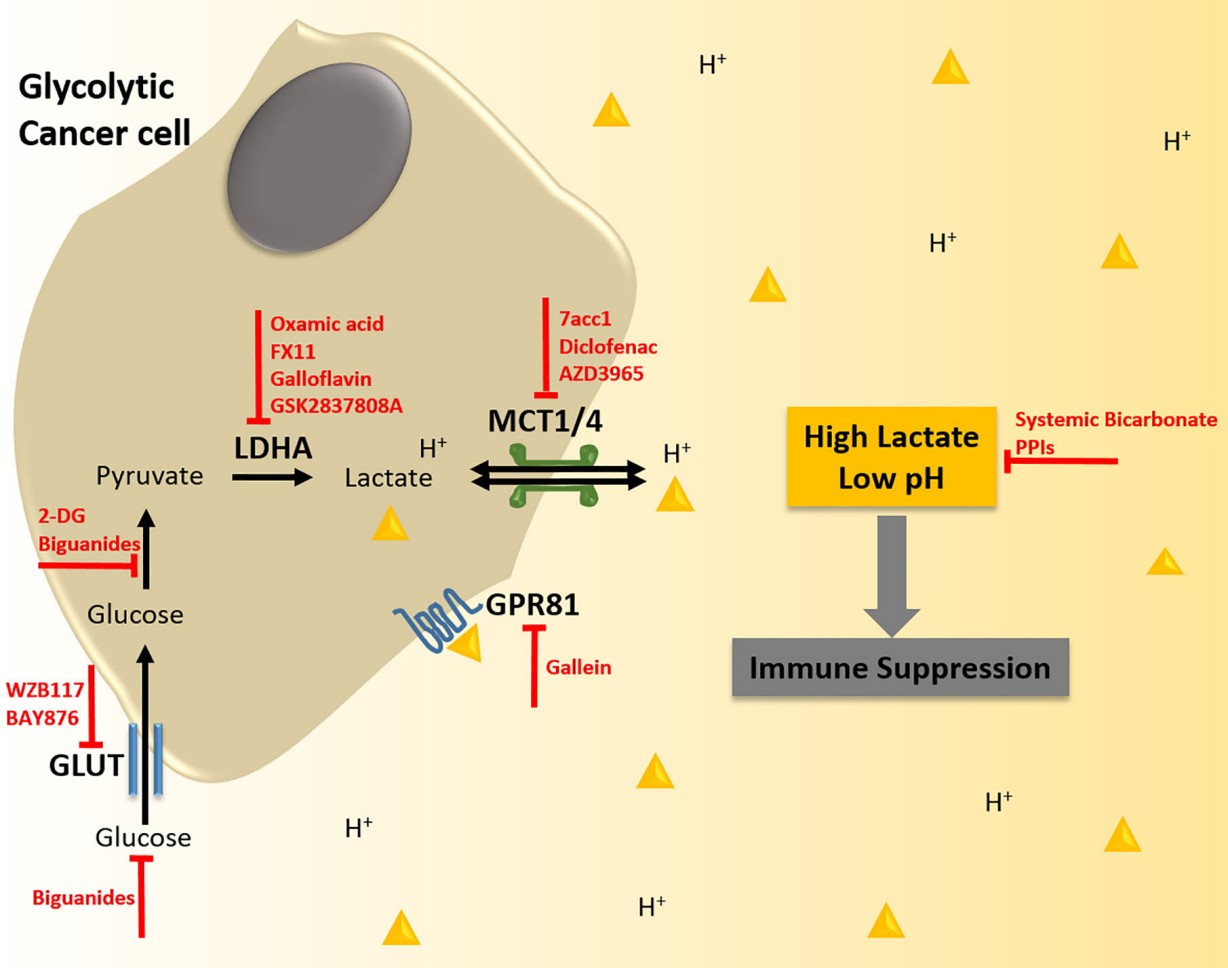
Strategies to target lactate biogenesis and acidosis to enhance immunotherapy response in triple negative breast cancer (TNBC). TNBC tumor cells display enhanced rates of glycolysis. This metabolic phenotype is supported by the increased expression of glucose transporters (GLUTs) that import glucose into the cell, and of lactate dehydrogenase A (LDHA) that converts the glycolytic intermediate pyruvate into lactate. The augmented production of lactate in TNBC tumors is also associated with higher expression of monocarboxylate transporters (MCTs), which shuttle lactate coupled to protons (H^+^) out of the tumor cell resulting in excessive levels of lactic acid in the tumor microenvironment (TME) and reduced pH. Lactate acidosis in the TME creates an immunosuppressive milieu, which can antagonize the efficacy of immunotherapy. Thus, anti-metabolic strategies could alleviate lactic acid-induced immunosuppression and potentiate immunotherapy such as Adoptive T cell therapy (ACT), Chimeric Antigen Receptor T cell (CAR-T) therapy, and Immune Checkpoint Blockade (ICB), thereby synergistically inhibiting tumorigenesis. Potential strategies to abrogate lactate biogenesis and acidosis include specific targeting of GLUTs, LDHA, MCTs, and the lactate-receptor GPR81 with small molecule inhibitors, inhibition of glucose-pyruvate conversion, systemically lowering the availability of glucose by treatment with biguanides, and buffering the intra-tumoral pH with bicarbonate therapy or proton pump inhibitors (PPIs).

Metabolic reprogramming, lactate accumulation, and metabolic competition promotes immunosuppression in the tumor microenvironment and is thus capable of modulating the efficacy of immunotherapy. In line with this, elevated tumor glycolysis has been reported as a negative prognostic indicator in immunotherapy. For instance, melanoma tumors that are refractory to adoptive T cell therapy (ACT) display high rates of glycolysis and reduced TILs (98). Likewise, elevated baseline serum LDH levels are associated with limited clinical benefit from ICB treatment in several tumor types including TNBC (126–128). Nevertheless, ICB therapy has shown promising results in certain glycolytic cancers such as TNBC. The success of ICB in these tumors might be in part the result of an anti-metabolic effect on both tumor and immune cells. PD-L1 expression on tumor cells has been found to support tumor glycolysis *via* the activation of mTOR/Akt pathway (95). Hence, anti-PD-L1 ICB may not only release T cell inhibition but also impair tumor glycolysis, lactate production, and metabolic competition between immune and tumor cells. In addition, ICB therapy may induce a shift in the metabolic needs of cytotoxic immune cells. While activation of T cell effector function relies on glycolysis, ligation or inhibition of PD-1 on T cells inhibits glycolysis and instead switches to fatty acid oxidation, which is crucial for maintaining T cell memory function and long-term anti-tumor activity (129). This phenomenon further allows T memory cells to thrive by reducing their dependence on glucose and hence avoiding metabolic competition within the tumor microenvironment. In addition to harnessing the anti-metabolic potential of ICBs, there is also evidence supporting exploiting tumor acidity to improve treatment response to ICB. In a preclinical study, Johnston et al. show that activation of the checkpoint molecule V-domain immunoglobulin suppressor of T cell activation (VISTA) was more prominent under acidic conditions such as those found in highly glycolytic tumors with lactate acidosis (130). Blocking VISTA with a monoclonal antibody could reverse the immunosuppressive activity, particularly in combination with anti-PD-1, leading to enhanced T cell infiltration, dampened expression of checkpoint receptors on T cells (PD-1, LAG-2, and TIM-3), and subsequent increased anti-tumor activity in MC38 colorectal carcinoma-bearing mice. Further investigation in mice and cynomolgus macaque models showed that acidic pH-selective anti-VISTA antibodies preferentially accumulated in tumor tissue, suggesting minimal risk of off-target effects even though VISTA is expressed by leukocytes. Therefore, combining immunotherapy with strategies to either mitigate tumor glycolysis and lactate levels or specifically render immune cells resistant to the hostile tumor microenvironment may prove advantageous in improving therapeutic response in TNBCs (**Figure 2**). Here, we speculate on the potential of anti-metabolic strategies to enhance the efficacy of immunotherapy and their associated challenges.

### Targeting Molecular Mediators of the Warburg Phenotype

Inhibiting tumor glucose uptake, glycolysis and lactate transport have been proposed to reduce both tumor growth and immunosuppression, thus rendering these strategies compelling candidates for combination therapy. Specific and potent inhibitors of the GLUT transporters have been identified and investigated for their anti-tumor activity in pre-clinical studies (131). For instance, BAY-876 and WZB117 GLUT-1 inhibitors have shown anti-proliferative effects in breast tumor cells (132). In particular, a subset of TNBC tumors expressing the retinoblastoma (Rb) tumor suppressor with high glycolytic activity and low OXPHOS are sensitive to GLUT1 inhibition with BAY-876 (133). Since GLUTs are ubiquitously expressed, the impact of their inhibition at peripheral organs still needs to be well documented. Treatment with 2-DG, a non-metabolizable glucose analog and inhibitor of hexokinase, restricts tumor glycolysis and growth. Furthermore, combining 2-DG with mitochondria-targeting agents synergistically eradicates metabolic plasticity and enhances tumor regression in a TNBC xenograft model (134). Inhibition of glycolysis by 2-DG also dampens tumor cell production of G-CSF and GM-CSF in TNBC models, thus restricting MDSC development (86). Similarly, Dichloroacetate (DCA), an agent that shifts metabolic flux from glycolysis to OXPHOS, has shown efficacy in restricting tumor growth, specifically in tumor types with dysfunctional mitochondrial function such as TNBC (135). However, glycolysis inhibition beyond tumor cells could adversely affect T cell activation and trigger the induction of immunosuppressive Tregs and M2 TAMs (136). Notably, it has been argued that inhibiting glycolysis could drive T cells to a memory phenotype, a silver lining for long-term anti-tumor response (137, 138). Another approach to reduce glucose uptake in tumor cells and improve ICB response involves limiting glucose availability using anti-hyperglycemic biguanide drugs such as metformin and phenformin and glucose-limiting dietary interventions (139, 140). In murine B16 and MC38 melanoma models, combining anti-PD-1 treatment with metformin significantly reduced tumor growth by metabolic remodeling, reduced tumor hypoxia and improved T cell infiltration and function, as compared to either treatment alone (141). This combination treatment is currently under investigation in human clinical trials for advanced melanoma and non-small cell lung cancer (NSCLC) (NCT04114136) (142). Further, metformin can induce PD-L1 glycosylation and degradation thereby enhancing CTL activity and improving the efficacy of immunotherapy (143). While these studies appear promising, it should be noted that the effects of systemic interventions are pleiotropic and require careful investigation for off-target effects in combination with immunotherapy.

Targeting lactate dehydrogenases, in particular LDHA, offers another lucrative approach to alter the balance in tumor metabolic needs and to shape the composition and orientation of the immune microenvironment. Treatment with LDHA inhibitors such as oxamic acid, FX11, galloflavin, and 1-(phenylseleno)-4-(trifluoromethyl) benzene (PSTMB) demonstrate anti-proliferative effects in TNBC and other cancer models (144–146). Furthermore, treatment with oxamic acid enhanced CTL IFN-γ production, promoted DC differentiation, improved TNF secretion in monocytes, and abrogated M2 macrophage polarization in *in vitro* co-culture models (78, 89, 109, 120). Similarly, LDHA knockdown enhanced T cell infiltration and reduced the number of TAMs, leading to improved survival in the murine 4T1 TNBC model (147). LDHA depletion was also found to decrease MDSC development, improve NK cell cytotoxicity and hence, enhance anti-tumor immune response in multiple murine tumor models (81, 86). Interestingly, LDHA and PD-L1 are both negatively regulated by miR-34a and correlate with poor prognosis in TNBC, providing rationale for combining ICB therapy with LDHA inhibition (148). In accordance, lactate-mediated upregulation of PD-L1 has been observed in lung cancer and melanoma (74, 149). LDHA abrogation in the murine B16F10 melanoma model improved response to anti-PD-1 treatment, accompanied by an increase in tumor infiltration of CD8+ T cells and NK cells, increase in production of IFN-γ and granzyme B, and decrease in Treg infiltration (149). Moreover, combining the LDHA inhibitor GSK2837808A with ACT in a syngeneic murine melanoma model profoundly improved the anti-tumor response and survival compared to either LDHA inhibition or ACT alone (98). However, LDHA inhibitors have not yet successfully transitioned into clinical trials due to limited membrane permeability and on-/off-target toxicity (150). Moreover, the impact of LDHA inhibition on the viability and cytotoxicity of TILs needs to be explored extensively considering their need of glycolysis for activation.

As lactate transporters also play a key role in metabolic adaptation, their inhibition may provide another way to induce a metabolically favorable TME for immune cells. Indeed, MCT1/4 inhibition improved CD8+ T cell functionality *in vitro*, and the MCT4 inhibitor 7acc1 enhanced NK cell cytotoxicity and attenuated tumor growth in the murine 4T1 TNBC model (147). Although the MCT1/2 inhibitor AR-C155858 did not show any effect on tumor growth in the murine 4T1 TNBC model (151), its analogue AZD3965 is currently being assessed in a phase I clinical trial in solid tumors, diffuse large B cell lymphoma, and Burkitt’s lymphoma (NCT01791595). Interestingly, the non-steroidal anti-inflammatory drug (NSAID) diclofenac was found to be a potent inhibitor of MCT1/4 and to reduce intra-tumoral lactate levels, concomitant with inhibition of tumor growth and Treg infiltration in a glioma model (152). A more recent study explored the molecular mechanisms of diclofenac-mediated tumor inhibition using various co-culture and murine tumor models (153). Of note, the authors found that diclofenac alone or in combination with the MCT1/2 inhibitor AZD3965 didn’t negatively impact T cell viability and effector functions despite reducing the glycolytic activity of the cells due to their metabolic adaptability and shift to OXPHOS. Treatment of 4T1 cells with diclofenac reduced the expression of MCT1 and LDHA, while increasing major histocompatibility complex (MHC)-I and MHC-II surface expression. Furthermore, diclofenac increased tumor infiltration of activated T cells and IFN-γ+ NK cells and delayed tumor growth in the 4T1 TNBC mouse model. Combining diclofenac treatment with single anti-PD-1 or dual checkpoint blockade (anti-PD-1 plus anti-CTLA-4) inhibited tumor growth and increased treatment response in two murine models, 4T1 TNBC and B16F10 melanoma. Although encouraging, the efficacy and safety profile of this combination treatment remains to be confirmed. Recent pre-clinical reports have also hypothesized that pharmacological blockade of GPR81 may prove advantageous in improving response to immunotherapy by enhancing DC antigen presentation and dampening PD-L1 expression in lactate-rich environments (72). Blocking GPR81-mediated lactate signaling by gallein decreased the frequency of intra-tumoral Tregs and delayed tumor growth in the murine 4T1 model (85). Of importance, *Gpr81*-null mice did not exhibit any detrimental phenotypes, indicating that off-target effects of targeting GPR81 may be minimal. Nevertheless, high affinity GPR81 inhibitors are yet to be identified.

Collectively, the promising findings of the aforementioned studies suggest that inhibition of glycolysis through LDH and/or MCT inhibition may improve treatment response to ICB. However, it is paramount to minimize the risk of off-target effects since it has become evident that immune cells and tumor cells exploit common metabolic mechanisms and display an overlap in expression of the major players in lactate biogenesis and export. Moreover, ubiquitous expression of candidate targets such as LDHA and MCT1/4 in normal tissues necessitates extensive risk assessment of small-molecule inhibitors before considering combination with immunotherapy.

### Targeting of Metabolic Lactate Acidosis

One important aspect of lactic acid-mediated immunosuppression within solid tumors is the detrimental effect of the accompanying acidosis. Thus, repurposing drugs that modulate systemic metabolism may represent an opportunity to improve the response to immunotherapy. Oral bicarbonate therapy has been extensively used to treat metabolic acidosis associated with chronic kidney disease. Pre-clinical evidence for its utility in cancer therapy was provided by a study that demonstrated its ability to buffer intra-tumoral pH and inhibit tumor growth, concomitant with increased CD8+ T cell infiltration in murine melanoma and pancreatic tumor models (154). In addition, oral bicarbonate improved NK cell infiltration and IFN-γ production in a murine lymphoma model, resulting in delayed tumor growth (104). Moreover, combining bicarbonate therapy with anti-PD-1 or anti-CTLA-4 checkpoint blockade or ACT improved tumor regression in comparison to either treatment alone in murine cancer models. The efficiency of bicarbonate therapy to improve cancer immunotherapy response in humans remains to be confirmed.

Likewise, multiple proton pump inhibitors (PPIs), commonly used as antacids, are being clinically investigated for their ability to modulate intra-tumoral pH in solid tumors (155). PPIs can be administered as prodrugs that are activated in low pH microenvironments to subsequently interact with and inhibit the activity of H+/K+-ATPase, thus making them well-tolerated and safe even at high doses. Treatment with the PPI Esomeprazole showed an increase in tumor pH and improved effector function of TILs in B16 melanoma xenografts without increasing activation of T cell subsets isolated from peripheral organs (101). Combining PPI treatment with ACT enhanced the anti-tumor effect and overall survival. Surprisingly, several clinical studies assessing the efficacy of PPIs in combination with anti-PD-1/PD-L1 therapy have shown either no effect or an adverse effect on ICB response in melanoma and NSCLC patients (156, 157). It is important to note here that individual PPIs have different acid neutralizing abilities, and therefore reduce the diversity of the gut microbiome at varying degrees, which in turn is known to affect the response to ICB. It may be prudent to assess gut microbiome diversity and consider history of antibiotics use prior to treatment of cancer patients with acidosis-reducing agents and ICB.

### Targeting Immunometabolism

In addition to reducing the hostility of the TME, an alternative strategy involves engineering autologous T cells to optimize metabolic adaptation and confer more resistance to glucose-limiting, lactate-rich conditions (158, 159). For example, overexpression of phosphoenolpyruvate carboxykinase 1 (PCK1), a regulator of gluconeogenesis, could increase the production of phosphoenolpyruvate (PEP) that is required for sustained T cell effector function (160). Mechanistically, PEP suppresses sarco/endoplasmic reticulum Ca2+-ATPase (SERCA) activity in glucose-deprived T cells and improves Ca2+ flux and NFAT signaling required for T cell cytotoxicity. Adoptive transfer of PCK1-overexpressing T cells into the B16 murine melanoma model demonstrated improved production of CD4+ T cell derived IFN-γ and increased expression of the M1 macrophage CD86 marker on TAMs, collectively suppressing tumor growth and improving survival. Similarly, overexpression of PPAR-gamma coactivator 1α (PGC1α) restores mitochondrial dysfunction and biogenesis in tumor-infiltrating T cells supporting enhanced anti-tumor efficacy in B16 melanoma mice (161). Engineering chimeric antigen receptor (CAR) T cells to include the 4-1BB/CD137 signaling domain promotes the development of CD8+ memory T cells with an OXPHOS phenotype that may be beneficial to withstand metabolic competition within glycolytic TNBC tumors, as opposed to inclusion of the CD28 domain that induces a glycolytic phenotype in T cells (162). Thus, metabolic preconditioning of immune cells by ACT can enhance their persistence and effector function within the TME.

## Conclusion

Anti-cancer therapy has proven most effective in combinatorial settings, as tumors can quickly adapt to extrinsic cues. As such, improving long-term response rates to immunotherapy requires both direct and indirect modulation of anti-tumor immunity through a better understanding of the tumor-immune cell interface. One of the many unanswered questions pertains to the feasibility of targeting tumor cell metabolism without negatively affecting immune cell metabolism in order to enhance immunotherapy response. Ideally, metabolic interventions should aim to target unique vulnerabilities of tumor cells, without dampening anti-tumor immune function and eliciting undesirable effects on peripheral organs—the paradigm of “cellular selectivity based on demand” (136). Although direct intra-tumoral injection of anti-metabolic agents and immunotherapy in solid tumors such as breast tumors is lucrative in principle (163), the effect of this mode of delivery on normal mammary function requires investigation. This is particularly relevant in the case of lactating breast cancer patients since mammary epithelial cells considerably rely on glucose uptake for lactose biosynthesis. In this regard, gaining insight into the potential role of tumor-specific antigens, such as LDHC, in cancer metabolism could aid the development of specific inhibitors to circumvent the risk of adversely impacting normal cells. Alternatively, small molecules inhibiting lactate biogenesis or export could be delivered specifically to tumor cells using polymeric nanocarriers that are responsive to tumor-specific cues such as pH-sensitive nanoparticles that could facilitate drug delivery to lactate-rich tumor microenvironments (164).

The timing and sequence of the combinatorial approach also requires optimization. For instance, targeting glycolysis or lactate transport in tumor cells prior to ACT may reduce unfavorable effects on immune function. As anti-metabolic therapies are developed for cancer treatment, their efficacy and effects on anti-tumor immune response requires close monitoring. Moreover, the impact of metabolic heterogeneity as observed between TNBC subtypes in addition to intra-tumoral heterogeneity, on the response to immunotherapy and combinatorial approaches requires in-depth investigation (165). Lastly, well-designed interventional studies examining intra-tumoral or systemic biomarkers to enable stratification of TNBC patients that may benefit from combining immunotherapy with anti-metabolic strategies are essential. Such biomarkers could include genetic mutations or variants that have been associated with metabolic reprogramming in TNBC (166) such as mutant p53 (19), *BRCA1* mutations (167, 168), c-Myc amplification (169), and Rb expression (133). Notably, investigation of congenital lactic acidosis has identified distinct genetic variants that result in defective mitochondrial function and drive the pathogenesis of the disease (170) and hence, assessment of the effect of these genetic alterations on tumor metabolic phenotype would be of interest in the search for prognostic biomarkers.

Although this review focuses on TNBC, it is important to note that endocrine-resistant luminal breast cancer and trastuzumab-resistant HER2+ breast cancer also exhibit metabolic reprogramming, whereby the glycolysis rate and associated lactate acidosis are increased (17, 171–173). Hence, in analogy with the observed metabolic changes in TNBC, it is likely that the glycolytic TME in treatment-resistant luminal and Her2+ tumors could disrupt immune surveillance and negatively affect the response to immunotherapy. However, in comparison to TNBC tumors, HER2+ and in particular luminal or hormone receptor positive tumors display a significantly lower infiltration of TILs (174), potentially explaining the inferior efficacy of immunotherapy in these tumors. Moreover, in contrast to TNBC, the presence of a sparse TIL infiltration in hormone receptor positive tumors has been associated with worse clinical outcome (175, 176). These observations highlight the importance of considering the immune landscape characteristics of each breast cancer subtype as well as the auxiliary role of lactate acidosis in modulating anti-tumor immunity in order to predict immunotherapy response. Immunotherapy strategies such as ACT and CAR-T therapy have shown great potential to improve the immune permissiveness of luminal breast tumors (177), and could likely be used in combination with anti-metabolic strategies in endocrine-resistant tumors.

To conclude, identification and development of the next generation of immune-based therapeutic approaches that can improve the intra-tumoral metabolic landscape and hence augment the anti-tumor response is gaining interest and necessitates extensive research in this direction.

## Author Contributions

AN conceived and drafted the manuscript and designed the figures and table. JD conceived and critically revised the manuscript. All authors contributed to the article and approved the submitted version.

## Funding

This work was supported by a grant from the Qatar Biomedical Research Institute (#VR94), awarded to JD.

## Conflict of Interest

The authors declare that the research was conducted in the absence of any commercial or financial relationships that could be construed as a potential conflict of interest.
